# Supplementary material for: Heparin-based hydrogel scaffolding alters the transcriptomic profile and increases the chemoresistance of MDA-MB-231 triple-negative breast cancer cells
Source: Biomater Sci. 2020 Feb 13;8(10):2786–96. doi: 10.1039/c9bm01481k (PMC7497406; doi:10.1039/c9bm01481k)
Supplement: Supplementary file 2 [file BM-008-C9BM01481K-s002.zip › Supplementary File 4/HP-B hydrogel + EGF (s)vControl/Pathways/my_analysis.Gsea.1545201170987/HALLMARK_BILE_ACID_METABOLISM.html]

Details for gene set HALLMARK\_BILE\_ACID\_METABOLISM[GSEA]

|  || Dataset | expr.class.cls#HYDEGF\_versus\_CONTROL.class.cls#HYDEGF\_versus\_CONTROL\_repos |
| Phenotype | class.cls#HYDEGF\_versus\_CONTROL\_repos |
| Upregulated in class | CONTROL |
| GeneSet | HALLMARK\_BILE\_ACID\_METABOLISM |
| Enrichment Score (ES) | -0.4419837 |
| Normalized Enrichment Score (NES) | -1.8456464 |
| Nominal p-value | 0.0 |
| FDR q-value | 7.905337E-4 |
| FWER p-Value | 0.007 |
Table: GSEA Results Summary

  

Fig 1: Enrichment plot: HALLMARK\_BILE\_ACID\_METABOLISM      
 Profile of the Running ES Score & Positions of GeneSet Members on the Rank Ordered List

  

| PROBE | DESCRIPTION (from dataset) | GENE SYMBOL | GENE\_TITLE | RANK IN GENE LIST | RANK METRIC SCORE | RUNNING ES | CORE ENRICHMENT || 1 | ABCA5 | na |  |  | 510 | 2.107 | 0.0009 | No |
| 2 | LIPE | na |  |  | 526 | 2.095 | 0.0275 | No |
| 3 | EFHC1 | na |  |  | 802 | 1.816 | 0.0369 | No |
| 4 | NEDD4 | na |  |  | 2101 | 1.239 | -0.0148 | No |
| 5 | PEX16 | na |  |  | 2236 | 1.200 | -0.0061 | No |
| 6 | MLYCD | na |  |  | 2419 | 1.147 | -0.0006 | No |
| 7 | SLC23A2 | na |  |  | 2699 | 1.081 | -0.0010 | No |
| 8 | SLC23A1 | na |  |  | 2940 | 1.018 | -0.0002 | No |
| 9 | PEX6 | na |  |  | 3525 | 0.900 | -0.0190 | No |
| 10 | HACL1 | na |  |  | 3545 | 0.895 | -0.0083 | No |
| 11 | BCAR3 | na |  |  | 3587 | 0.886 | 0.0012 | No |
| 12 | SLC27A5 | na |  |  | 3870 | 0.835 | -0.0026 | No |
| 13 | PIPOX | na |  |  | 4027 | 0.808 | -0.0002 | No |
| 14 | TFCP2L1 | na |  |  | 4191 | 0.783 | 0.0015 | No |
| 15 | GSTK1 | na |  |  | 4536 | 0.729 | -0.0070 | No |
| 16 | NR3C2 | na |  |  | 4589 | 0.722 | -0.0002 | No |
| 17 | FDXR | na |  |  | 4868 | 0.674 | -0.0059 | No |
| 18 | DHCR24 | na |  |  | 4964 | 0.657 | -0.0023 | No |
| 19 | HSD17B6 | na |  |  | 5155 | 0.629 | -0.0040 | No |
| 20 | PFKM | na |  |  | 5189 | 0.622 | 0.0024 | No |
| 21 | ABCG4 | na |  |  | 5239 | 0.614 | 0.0079 | No |
| 22 | RXRA | na |  |  | 5268 | 0.611 | 0.0144 | No |
| 23 | PAOX | na |  |  | 5712 | 0.544 | -0.0016 | No |
| 24 | CYP46A1 | na |  |  | 5724 | 0.543 | 0.0049 | No |
| 25 | PEX11G | na |  |  | 6514 | 0.435 | -0.0307 | No |
| 26 | ABCA2 | na |  |  | 6951 | 0.379 | -0.0485 | No |
| 27 | PEX26 | na |  |  | 7694 | 0.280 | -0.0837 | No |
| 28 | BMP6 | na |  |  | 7945 | 0.250 | -0.0935 | No |
| 29 | DIO2 | na |  |  | 8419 | 0.199 | -0.1156 | No |
| 30 | ABCA3 | na |  |  | 8759 | 0.156 | -0.1313 | No |
| 31 | LONP2 | na |  |  | 9141 | 0.110 | -0.1498 | No |
| 32 | ABCD1 | na |  |  | 9269 | 0.094 | -0.1552 | No |
| 33 | SLCO1A2 | na |  |  | 9330 | 0.085 | -0.1573 | No |
| 34 | PEX1 | na |  |  | 10323 | -0.026 | -0.2088 | No |
| 35 | GCLM | na |  |  | 10721 | -0.067 | -0.2287 | No |
| 36 | PXMP2 | na |  |  | 10808 | -0.079 | -0.2322 | No |
| 37 | ACSL1 | na |  |  | 11022 | -0.109 | -0.2419 | No |
| 38 | HSD3B7 | na |  |  | 11035 | -0.112 | -0.2410 | No |
| 39 | GNMT | na |  |  | 11056 | -0.115 | -0.2406 | No |
| 40 | SLC29A1 | na |  |  | 11581 | -0.172 | -0.2657 | No |
| 41 | AMACR | na |  |  | 11754 | -0.196 | -0.2722 | No |
| 42 | ATXN1 | na |  |  | 12145 | -0.236 | -0.2895 | No |
| 43 | ABCD3 | na |  |  | 12429 | -0.275 | -0.3007 | No |
| 44 | PEX11A | na |  |  | 12605 | -0.299 | -0.3059 | No |
| 45 | RETSAT | na |  |  | 12706 | -0.315 | -0.3070 | No |
| 46 | AR | na |  |  | 12810 | -0.330 | -0.3081 | No |
| 47 | NR1I2 | na |  |  | 12945 | -0.335 | -0.3107 | No |
| 48 | CROT | na |  |  | 13059 | -0.351 | -0.3120 | No |
| 49 | IDH2 | na |  |  | 14028 | -0.492 | -0.3562 | No |
| 50 | GNPAT | na |  |  | 14869 | -0.614 | -0.3921 | No |
| 51 | SCP2 | na |  |  | 15058 | -0.644 | -0.3935 | No |
| 52 | OPTN | na |  |  | 15986 | -0.816 | -0.4313 | Yes |
| 53 | SLC22A18 | na |  |  | 15992 | -0.817 | -0.4209 | Yes |
| 54 | PEX12 | na |  |  | 16113 | -0.849 | -0.4160 | Yes |
| 55 | FADS2 | na |  |  | 16193 | -0.868 | -0.4088 | Yes |
| 56 | SLC35B2 | na |  |  | 16353 | -0.911 | -0.4052 | Yes |
| 57 | ALDH9A1 | na |  |  | 16400 | -0.923 | -0.3955 | Yes |
| 58 | PEX13 | na |  |  | 16767 | -1.016 | -0.4014 | Yes |
| 59 | FADS1 | na |  |  | 16789 | -1.024 | -0.3891 | Yes |
| 60 | NPC1 | na |  |  | 17307 | -1.184 | -0.4006 | Yes |
| 61 | ACSL5 | na |  |  | 17434 | -1.228 | -0.3912 | Yes |
| 62 | PECR | na |  |  | 17761 | -1.363 | -0.3904 | Yes |
| 63 | NUDT12 | na |  |  | 17794 | -1.380 | -0.3740 | Yes |
| 64 | ABCA1 | na |  |  | 17887 | -1.432 | -0.3600 | Yes |
| 65 | ISOC1 | na |  |  | 17943 | -1.462 | -0.3438 | Yes |
| 66 | IDI1 | na |  |  | 18098 | -1.547 | -0.3316 | Yes |
| 67 | ABCA6 | na |  |  | 18184 | -1.597 | -0.3151 | Yes |
| 68 | PEX19 | na |  |  | 18274 | -1.656 | -0.2981 | Yes |
| 69 | CYP27A1 | na |  |  | 18279 | -1.661 | -0.2766 | Yes |
| 70 | PEX7 | na |  |  | 18285 | -1.664 | -0.2550 | Yes |
| 71 | PNPLA8 | na |  |  | 18529 | -1.858 | -0.2434 | Yes |
| 72 | APOA1 | na |  |  | 18534 | -1.861 | -0.2193 | Yes |
| 73 | HSD17B11 | na |  |  | 18643 | -1.977 | -0.1991 | Yes |
| 74 | CAT | na |  |  | 18668 | -2.014 | -0.1740 | Yes |
| 75 | IDH1 | na |  |  | 18690 | -2.035 | -0.1484 | Yes |
| 76 | PHYH | na |  |  | 18766 | -2.139 | -0.1243 | Yes |
| 77 | SOD1 | na |  |  | 18910 | -2.370 | -0.1008 | Yes |
| 78 | HSD17B4 | na |  |  | 19012 | -2.605 | -0.0720 | Yes |
| 79 | CYP39A1 | na |  |  | 19068 | -2.796 | -0.0383 | Yes |
| 80 | PRDX5 | na |  |  | 19161 | -3.438 | 0.0019 | Yes |
Table: GSEA details [plain text format]

  

Fig 2: HALLMARK\_BILE\_ACID\_METABOLISM      
 Blue-Pink O' Gram in the Space of the Analyzed GeneSet

  

Fig 3: HALLMARK\_BILE\_ACID\_METABOLISM: Random ES distribution      
 Gene set null distribution of ES for **HALLMARK\_BILE\_ACID\_METABOLISM**
